# The Prevalence of Potential Prescribing Omissions for Antiplatelets and Statins in Older Adults With Atherosclerotic Cardiovascular Disease

**DOI:** 10.7759/cureus.47540

**Published:** 2023-10-23

**Authors:** Samah Alshehri, Mohannad Alshibani, Ghazwa Krayem, Solafa Noorsaeed, Abdulmohsen Alghamdi, Sara Alotaibi, Orjwan Khayat, Abdulhamid Althagafi

**Affiliations:** 1 Clinical Pharmacy, King Abdulaziz University, Jeddah, SAU; 2 Pharmacy Practice, King Abdulaziz University, Jeddah, SAU; 3 Pharmacy Practice, Princess Nourah bint Abdulrahman University, Riyadh, SAU; 4 Medicine and Surgery, King Abdulaziz University Faculty of Medicine, Jeddah, SAU; 5 Obstetrics and Gynecology, King Fahad Medical City, Riyadh, SAU

**Keywords:** antiplatelet, statins, underprescribing, omission, older adults

## Abstract

Background

The Screening Tool to Alert to Right Treatment (START) criteria for older adults was developed to recognize potential prescribing omissions (PPOs) of clinically indicated medications. According to these criteria, statins and antiplatelets should be prescribed for older adults with a documented history of coronary artery, cerebral, or peripheral vascular disease unless contraindicated.

Aim

This study aimed to investigate physicians’ adherence to START criteria and identify the prevalence of PPO, considering the prescription of statins and antiplatelets in older patients with a history of coronary artery, cerebral, or peripheral vascular disease.

Methods

In this single-center, cohort, retrospective study, patients aged >65 years with a history of coronary artery, cerebral, or peripheral vascular disease were included. The prevalence of PPO for statins and antiplatelet therapy was identified. This study was guided by the screening Tool of Older Persons' Prescriptions (STOPP)/START criteria published in 2016.

Results

A total of 244 patients with a history of coronary, cerebral, or peripheral vascular disease were included in this study. Statin use was appropriately observed in 131/220 (59.5%) while antiplatelets were appropriately observed in 219/237 (92.4%). Therefore, the PPO for statins and antiplatelets was 40.5% and 7.6%, respectively.

Conclusion

The results of this study identified that the prevalence of PPO for statins and antiplatelets for older adults was high. We encourage prescribers to utilize tools such as Beer’s criteria or STOPP/START, which may assist their decision in addition to their clinical judgment in weighing the benefit to the risk of starting or stopping antiplatelets or statins in older adult patients.

## Introduction

The total world population of individuals aged 60 years or over doubled from 382 million in 1980 to 962 million in 2017. This number is also projected to double by 2050 [1]. According to the General Authority of Statistics, the percentage of geriatrics in Saudi Arabia was estimated to be 3.2% in 2018. In contrast, the total population in Jeddah was 4,433,000 in 2018. It is estimated that the number of geriatrics in Jeddah was 140,777 during the same period [2]. Additionally, the United Nations predicted that the percentage of older adults in Saudi Arabia would continue to increase, making up 18.4% of the total population by 2050 [3]. This puts a burden on the healthcare system to ensure adequate services that meet this population’s needs. Even though some older adults are healthy, the majority are frail and require assistance and health care. Thus, it is essential to pay attention to the health of older adults [4].

Older adults’ body systems undergo several physiological, dynamic, and kinetic changes as they age, many of which require medication management to treat or prevent diseases among older people with multimorbidity. However, one of the most common concerns for older adults is the risk of polypharmacy, inappropriate prescribing (IP), or prescribing omission (PO). These issues may lead to adverse drug reactions (ADRs) and clinical complications [5]. Therefore, many effective tools have been developed to prevent IP among older adults, such as the Screening Tool of Older Person's Prescriptions (STOPP) and the Screening Tool to Alert to Right Treatment (START) criteria [6]. The STOPP/START criteria are arranged according to physiological systems, including cardiovascular and endocrine systems [7,8].

It has been reported that at least 68% of people aged 65 or older with diabetes die from cardiovascular diseases (CVDs), and 16% die from stroke. The risk of adults with diabetes dying from CVDs is 2 to 4 times higher than the risk for nondiabetic adults. Thus, the START criteria include potentially giving antiplatelets and statins to those aged 65 years or more with a documented history of coronary artery, cerebral, or peripheral vascular disease where no contraindications exist. A recent multicenter observational study reported the rate of potentially inappropriate prescribing (PIP) and potential prescribing omission (PPO) among older adults to be 54.4% and 44.5%, respectively [9]. However, no data about the rate of PIP and PPO according to STOPP/START criteria currently exist in Saudi Arabia. Thus, the aim of this study is to investigate adherence to START criteria and identify the prevalence of PPO for statins and antiplatelets for older adults with a documented history of coronary, cerebral, or peripheral vascular disease.

## Materials and methods

The study was a retrospective cohort that was carried out at a single-center, tertiary hospital located in Jeddah, Saudi Arabia. This study specifically targets individuals aged 65 years and above, who had documented histories of coronary, cerebral, or peripheral vascular diseases. This encompassed conditions like stroke, ischemic heart disease, myocardial infarction, aortic aneurysms, and peripheral artery disease.

Certain exclusions were made based on contraindications for statin usage. This encompassed individuals with abnormal creatinine kinase levels (exceeding 10 times the upper normal range), a history of liver injury attributed to statin use, liver cirrhosis, and a prior history of liver abscess. Additionally, patients with contraindications to antiplatelet medications were also excluded. This involved individuals with a history of major bleeding, intracerebral hemorrhage, and gastrointestinal bleeding. Additionally, medical records with poor documentation were excluded.

Detailed demographic data were collected, including information on sex, nationality, age, smoking habits, weight, height, and the number of prescribed medications. Furthermore, the medical records of the participants were used to retrieve their medical history related to cardiovascular diseases (CVDs), specific diagnoses, as well as the utilization of statins and antiplatelet drugs.

In the evaluation of medication administration, a judicious reliance on the STOPP/START criteria was instituted. These criteria serve as a valuable framework for physicians, offering systematic guidance in the assessment of the appropriateness of medications for elderly patients, within the framework of their current clinical status.

Statistical analyses were performed for each group using the Statistical Package for the Social Sciences (SPSS) software (Version 25.0. 2017, Armonk, NY: IBM Corp.). Categorical data were presented as frequencies, employing descriptive statistical analysis. The study received ethical approval from the Institutional Review Board at King Abdulaziz University, identified by IRB number 435-20, prior to any data collection. Given that no individuals were personally identified, participants' consent for the study was waived. The study adhered strictly to the principles outlined in the Declaration of Helsinki. Throughout the study, unwavering fidelity was maintained to the principles articulated in the Declaration of Helsinki, an ethical cornerstone that underpins the integrity and ethical conduct of medical research.

## Results

**Figure 1:**
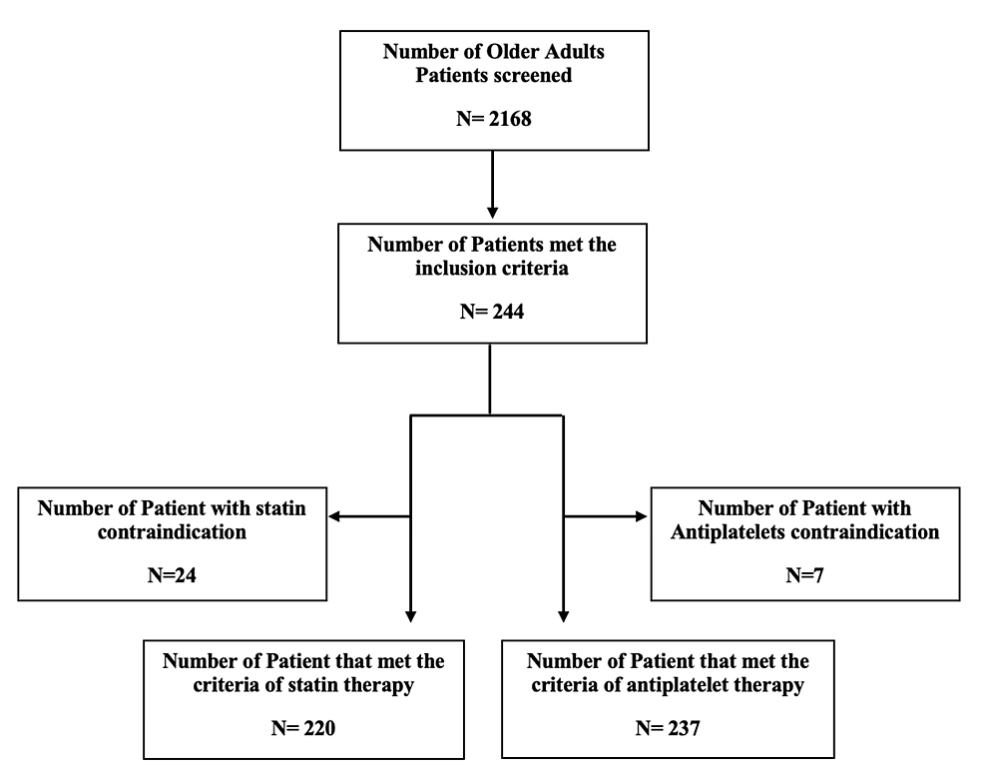
Patients' flowchart

A total of 2,168 older adult patients were admitted and screened for eligibility; 244 patients met the inclusion criteria (Figure 1). The baseline characteristics of the included patients are presented in Table 1. The mean age was 75 ± 7 years, and 144 (59.0%) were males. Among the included patients, 106 (43.4%) were Saudi. Regarding smoking history, 143 (58.6%) were smokers. Moreover, the median number of prescribed medications was six (4-11). Twenty-four patients (9.8%) had contraindications for statins; 7 had high creatinine kinase levels, 13 showed high levels in liver enzymes, 1 had liver cirrhosis, and 3 had a history of liver abscess. Moreover, only seven (2.9%) of the included subjects had contraindications for antiplatelets, four had a history of major bleeding, and three had intracerebral hemorrhages.

**Table 1:** Patients’ characteristics N: Number; %: percentage; SD: Standard Deviation; Kg: Kilogram; cm: Centimeter; IQR: Interquartile Range

Demographics		N (%) (N = 244)
Male, n (%)		144 (59%)
Age, mean ± SD, years		75 ±7
Saudi, n (%)		106 (43.4%)
Weight, mean ± SD, Kg		77.2 ± 19.6
Height, mean ± SD, cm		163.2 ± 13.8
Smoking History, n (%)		
	Smoker	143 (58.6%)
	Non-smoker	101 (41.4%)
Number of medications, median (IQR)		6 (4-11)
Cardiovascular disease		
	Ischemic heart disease	138 (56.6%)
	Stroke	73 (29.9%)
	Myocardial infarction	54 (22.2%)
	Peripheral artery disease	15 (6.2%)
	Aortic aneurysm	2 (0.9%)
Antiplatelets contraindication		7 (2.9%)
Statins contraindication		24 (9.8%)

Regarding statin therapy, 131 patients (59.5%) were using statins appropriately following the START/STOPP criteria, whereas statins were omitted in 40.5% of the patients, as presented in Table 2. Antiplatelets were prescribed appropriately according to the defined criteria in 219 patients (92.4%), whereas 18 patients (7.6%) were not on aspirin or clopidogrel, as shown in Table 3.

**Table 2:** Rate of omission for statins therapy N: Number; %: Percentage

Patients	N (%) (N = 220)
Patients on therapy	131 (59.5%)
Therapy omission	89 (40.5%)

**Table 3:** Rate of omission for antiplatelet therapy N: Number; %: Percentage

Patients	N (%) (N = 237)
Patients on therapy	219 (92.4%)
Therapy omission	18 (7.6%)

## Discussion

This study found that prescribers are underprescribing statins and antiplatelets in older adults in Saudi Arabia. The use of antiplatelets and statins in older adults is a cornerstone in the medical management of older adult patients. The omission of such essential therapies may put patients at an increased risk of CVDs or atherosclerotic cardiovascular diseases (ASCVDs). The underprescription of these medications may be attributed to patients’ or prescribers’ factors. Patients’ factors may include their preference, frailty, and/or life expectancy. While prescribers tend to cautiously use statins and antiplatelets due to insufficient data that support the use of primary statins as primary prevention in patients above the age of 75 or aspirin as primary prevention in patients above the age of 70 [10]. Moreover, the decision to use these agents for treatment is left to the prescribers’ discretion in weighing the benefit of these medications to their risk. Factors such as patients’ life expectancy, comorbidities, drug-drug interactions, or other risk factors may affect prescribers’ decisions.

The rate of PPO for appropriate medications from the patient’s medication regimens in this study was similar to previously reported numbers, ranging from 3.16% to 73.3% [8,11,12]. A multicenter prospective observational study of patients aged ≥ 65 years found the most frequent PPOs were CVD medications at 37.1% [9]. Another study, including patients aged ≥ 65 years, reported the most common PPOs to be statin therapy and antiplatelet agents, especially in patients with CVD risk and diabetes mellitus [13]. Similarly, a cross-sectional study, including 230 patients in Brazil, reported that the most common PPO was related to statin therapy at 29.8% and antiplatelets at 13.7% in older adult patients with diabetes mellitus who have coexisting cardiovascular risk factors [14]. In that study, dyslipidemia was found to be a protective factor for PPO in older adult patients [15].

Statin therapy has been proven to reduce ASCVD morbidity in older adults when used for secondary prevention. However, the American College of Cardiology/American Heart Association (ACC/AHA) defers the decision about using statins for primary prevention in adults aged > 75 to patients and physicians [16]. In our study, statins were omitted in 40.5% of older adults for whom statins were appropriate. This number was higher than previously reported rates of statins PPO [8,9]. A previous cross-sectional study, including 230 older adults with CVDs in a hospital in Ethiopia, reported that statin therapy was not initiated in 3.16% of patients [8]. Another multicenter prospective study reported the PPO of statins in older adults to be 6.7% [9]. Since the mean age of our study subjects was 75 ± 7, the high rate of statin omission may be attributed to prescribers’ concerns about the increased risk of myopathy, polypharmacy, and life expectancy.

Based on the emerging evidence about aspirin’s lack of mortality benefits in primary prevention [16], the ACC/AHA updated its recommendations about aspirin use. The AHA advises against the routine use of low-dose aspirin for the primary prevention of ASCVDs in adults above 70 years old [17]. The use of low-dose aspirin as a primary prevention increased with age [17]. At the same time, aging was associated with an increased risk of aspirin misuse as primary cardiovascular prevention [17,18]. In contrast, other studies have reported that diabetes, comorbidities (e.g., chronic obstructive pulmonary disease, DM), and the number of medications are associated with higher PPO rates [19]. Other patient factors related to PPO in older adults may include depression, polypharmacy, and female gender [12,20].

This study has several limitations. First, it was a single-centered retrospective study, and second, the sample size was small; both limited the study results’ generalizability. Third, the omission rate may be underrepresented because antiplatelets and statins are over-the-counter medications in Saudi Arabia; therefore, patients may obtain them from outside the hospital. Fourth, the study did not include patients with DM who may be eligible for antiplatelets and statin therapy. Finally, the study did not document other factors that might affect patients’ eligibility for antiplatelets such as allergies.

## Conclusions

The results of this study identified that the prevalence of PPO for statins and antiplatelets for older adults was high. We encourage prescribers to use their clinical judgment in weighing the benefits versus the risks of starting or stopping antiplatelets or statins in older adult patients. Utilizing tools such as Beer’s criteria or STOPP/START may assist their decision. Meanwhile, a larger prospective study needs to confirm our study findings and assess the consequences of potential IP or PPO on the health of older adults.
